# Financial protection in flux: longitudinal evidence on out-of-pocket spending among middle-aged and older Mexicans during the ‘Seguro Popular’–INSABI transition

**DOI:** 10.1093/heapol/czag059

**Published:** 2026-05-07

**Authors:** Jose Eduardo Cabrero-Castro, Marissa Gonzalez, Emma Aguila, Héctor Arreola-Ornelas, Michael Touchton, Felicia Marie Knaul, Alfonso Rojas-Alvarez

**Affiliations:** Department of Public Health Sciences, College of Health Sciences, The University of Texas at El Paso, 500 West University Avenue, El Paso, TX 79968, United States; Department of Public Health Sciences, College of Health Sciences, The University of Texas at El Paso, 500 West University Avenue, El Paso, TX 79968, United States; Sol Price School of Public Policy, University of Southern California, Ralph and Goldy Lewis Hall 311, Los Angeles, CA 90089-0626, United States; Unidad de Políticas Públicas en Salud, Instituto de Investigación sobre la Obesidad, Tecnológico de Monterrey, Av. Eugenio Garza Sada 2501 COL. Tecnolgico 64700 Monterrey, NL, Mexico; Escuela de Ciencias Sociales y Gobierno, Tecnológico de Monterrey, Av. Revolución 756, Mixcoac,Benito Juárez, 03700 Mexico City, Mexico; Fundación Mexicana Para la Salud (FUNSALUD), A.C., Anillo Perif. 4809, Arenal Tepepan, Tlalpan, 14610 Mexico City, Mexico; Tómatelo a Pecho, A.C., Mexico City, Mexico; Department of Political Science, College of Arts and Sciences, University of Miami, 1300 Campo Sano Avenue Coral Gables, FL 33146, United States; Institute for Advanced Study of the Americas, University of Miami, 1541 Brescia AveSuite 110 Coral Gables, FL 33146, United States; Fundación Mexicana Para la Salud (FUNSALUD), A.C., Anillo Perif. 4809, Arenal Tepepan, Tlalpan, 14610 Mexico City, Mexico; Tómatelo a Pecho, A.C., Mexico City, Mexico; Department of Medicine, David Geffen School of Medicine, University of California, Los Angeles (UCLA), Geffen Hall 885 Tiverton Drive, Los Angeles, CA 90095, United States; Escuela de Medicina y Ciencias de la Salud, Tecnológico de Monterrey, Facultad de Excelencia, Mexico City, Mexico; Department of Public Health Sciences, College of Health Sciences, The University of Texas at El Paso, 500 West University Avenue, El Paso, TX 79968, United States

**Keywords:** out-of-pocket expenditures, older adults, financial protection, health system reform, Mexico

## Abstract

Mexico is ageing rapidly, placing growing strain on health financing and long-term care systems. Older adults face a double burden: higher healthcare needs due to chronic conditions and multimorbidity, and limited or informal income in later life, leaving them highly exposed to out-of-pocket (OOP) spending. In 2020, the government replaced ‘Seguro Popular’ (SP) with the ‘Instituto de Salud para el Bienestar’ (INSABI) to strengthen financial protection, but its implications on older adults remain unclear. We analysed OOP among 13 616 individuals aged ≥50 years in the Mexican Health and Aging Study, interviewed in 2018 and 2021. Expenditures for hospitalizations, outpatient procedures, medical visits and medications in the previous 12 months were indexed to inflation and converted to 2021 US dollars. Tobit models estimated total and component OOP, including an interaction between insurance category (Uninsured; Social Security—IMSS/ISSSTE/PEMEX/Defence; SP 2018/INSABI 2021; Other) and survey year, adjusting for sociodemographic and health covariates. Between 2018 and 2021 the proportion of older adults reporting no health insurance tripled from 9.5% to 27.2%, while SP affiliation fell from 30.1% to 10.9%. Social Security beneficiaries spent substantially less than the uninsured on total OOP (about US$1 033 less in 2018 and US$539 less in 2021). SP in 2018 and INSABI in 2021 were also associated with lower OOP (−US$291 and −US$298 versus the uninsured, respectively). Only Social Security was associated with a statistically significant reduction in medication-related OOP. Overall, the transition from SP to INSABI coincided with a marked rise in reported uninsurance and persistently high OOP, particularly for medicines, the principal driver of financial burden among older adults. These findings highlight the fragility of recent health financing reforms and the need to ensure sustained, employment-independent financial protection for Mexico's ageing population.

Key messages

- Mexico's health financing reforms have unfolded in the context of rapid population ageing and high reliance on out-of-pocket spending, leaving adults aged 50 and older who have greater chronic disease burden and limited income security, particularly exposed to financial risk.
- Using longitudinal data from the Mexican Health and Aging Study (2018–21), we show that the replacement of Seguro Popular by INSABI was accompanied by a sharp contraction in reported public coverage among older adults, with the share of uninsured nearly tripling.
- Social Security consistently provided the strongest financial protection for older adults, especially for medicines, whereas Seguro Popular/INSABI offered only modest, incomplete reductions in out-of-pocket spending and did not significantly reduce medication-related expenditures.
- These findings suggest that rapid structural reforms, when implemented without robust institutional capacity, risk eroding financial protection for older people; health policy in Mexico and similar middle-income settings should prioritize employment-independent coverage, secure medicine supply chains, and pooled purchasing to protect ageing populations from rising out-of-pocket costs.

## Introduction

Mexico is ageing rapidly. Adults aged 60 years and older currently represent over 13% of the population, and projections from the National Population Council estimate this share will nearly double by 2050, reaching one in four Mexicans (CONAPO 2025). Population ageing brings a steep rise in multimorbidity, functional decline, and demand for continuous healthcare. Globally, the prevalence of multimorbidity among adults exceeds 37%, and in Latin America, older adults commonly live with two or more chronic conditions such as hypertension, diabetes, and arthritis (Chowdhury et al. 2023). In Mexico, this accumulation of chronic disease increases healthcare utilization and the need for medications, diagnostic procedures, and hospital care (Pérez-Zepeda 2024). Yet, as shown in recent analyses, many older Mexicans experience barriers in accessing public healthcare spanning availability, affordability, and perceived quality, which often drives them to seek private care and pay out-of-pocket (OOP) (Doubova et al. 2024, García-Hernández et al. 2024). Consequently, ageing and multimorbidity magnify the vulnerability of this population to financial hardship.

Financial risk protection, defined as the ability to access needed quality healthcare without undue financial hardship, is a cornerstone of universal health coverage and a key indicator of equitable, resilient health system performance (World Health Organization 2010, Rahman et al. 2022). However, OOP expenses in health remain a major source of financial vulnerability in many countries including Mexico. Despite decades of reform, OOP continues to finance approximately 40% of Mexico's current health expenses, which stands significantly higher than the OECD average of 13% (OECD 2023, World Health Organization 2025b).

This heavy reliance on private spending carries profound consequences for older adults, who experience higher morbidity and increased exposure to healthcare-related financial shocks (DeGraff et al. 2022). Complex care needs such as frequent healthcare visits, ongoing management of chronic diseases, and sustained medication use, combined with limited economic resources, exacerbate the risk of recurrent OOP and catastrophic health expenditures (Salinas-Escudero et al. 2022). Pharmaceuticals alone represent over 60% of household OOP in Mexico, disproportionately affecting older adults with multi-morbidities (OECD 2023).

Mexico's fragmented health system amplifies these vulnerabilities. It consists of employer-based contributory social security programs, government-sponsored insurance for unemployed and informal-sector workers, and private providers (Gómez-Dantés et al. 2024). Employer-based social security programs constitute a significant source of health coverage for the Mexican population, with the ‘Instituto Mexicano del Seguro Social’ (IMSS) and the ‘Instituto de Seguridad y Servicios Sociales de los Trabajadores del Estado’ (ISSSTE) serving as the two largest institutions (Knaul et al. 2023, Gómez-Dantés et al. 2024). IMSS insures formally employed private-sector workers and their dependents, who account for approximately 40% of the population, while ISSSTE covers federal employees and their families, reaching an additional 8% (Gómez-Dantés et al. 2024). Both institutions provide, at least on paper, comprehensive coverage, including preventive services, specialist care, hospitalizations, and medications, generally at little or no cost to beneficiaries. The rest of the population, predominantly informal workers, the unemployed, and rural residents comprising about half the population, lack access to pooled, public insurance and must rely on public sector health services and OOP payment (Gómez-Dantés et al. 2024).

In 2004, Mexico enacted a legal reform and introduced the System of Social Protection in Health, which incorporated ‘Seguro Popular’ (SP) as its operational arm to provide financial protection in health, with the aim of addressing these gaps and provide subsidized insurance to those outside the formal employment and social security systems (Knaul and Frenk 2005). Seguro Popular grew rapidly, peaking at approximately 56 million beneficiaries, or about 45% of the population, by 2016 (Colchero et al. 2022). Emphasizing prevention and primary care, SP offered coverage for 294 health interventions and more than 600 essential medicines through the Universal Catalogue of Health Services (CAUSES, in Spanish). It also included the Fund for Protection Against Catastrophic Expenditures, which by 2019 covered 66 high-cost health conditions (CONEVAL 2020). Together, these mechanisms expanded access to services and reduced catastrophic health expenditures for many previously uninsured Mexicans (Galárraga et al. 2010, Gómez-Dantés et al. 2024). Despite these notable successes, SP encountered ongoing criticism regarding inadequate coverage for certain health conditions, inconsistent service quality, and systemic inefficiencies linked to decentralized financing, uneven state-level implementation, bureaucratic complexities, and persistent OOP burdens for specialized care and medications (Laurell 2015, Colchero et al. 2022, Gómez-Dantés et al. 2024).

The Mexican government replaced the SP with the ‘Instituto de Salud para el Bienestar’ (INSABI) through a reform to the General Health Law enacted in late 2019 and implemented in January 2020, as part of a broader political and institutional reorientation of health policy for the population without social security. INSABI emerged from a political critique of segmented insurance arrangements and from the view that access to healthcare should be guaranteed directly by the state as a universal social right rather than mediated through enrolment in a specific insurance programme (Knaul et al. 2023). Under SP, financial protection for the uninsured operated through a decentralized scheme, in which federal funds were transferred to the states and implementation depended heavily on state-level arrangements. By contrast, INSABI was conceived as a more centralized model, with stronger federal control over financing, coordination, and regulation, and with the explicit aim of guaranteeing free health services, medicines, and associated supplies as a universal social entitlement rather than through enrolment in a separate insurance programme (González-Block 2017, Gómez-Dantés et al. 2024). Clarifying this policy orientation is important because these institutional differences likely shaped continuity of coverage, entitlement clarity, and the degree of financial protection experienced during the transition.

Complicating this transition was the concurrent onset of the COVID-19 pandemic, which lead to a 90% increase in catastrophic health expenses among households without insurance between 2018 and 2020 (Knaul et al. 2021, Serván-Mori et al. 2023). The abrupt policy shift led to unintended consequences; national insurance declined by approximately 17% between 2018 and 2020 (Knaul et al. 2023). By 2022, only 13% of the population reported affiliation with INSABI and private ambulatory care usage surged to 66%, suggesting a failure of public provision to meet population needs (Martínez-Valle 2025). In the face of these challenges, in May 2023 the federal government dissolved INSABI and reassigned its responsibilities to IMSS-‘Bienestar’, a service network independent from the social security insurance IMSS (Diario Oficial de la Federación 2023).

Examining OOP during the 2018–2021 transition from SP to INSABI provides key insight into the changes associated with these policies on the older adult, uninsured population. This interval marked Mexico's first attempt to replace a decentralized, state-financed scheme with a centrally funded model, revealing persistent challenges (drug supply disruptions, shrinking coverage, and growing reliance on private providers) that IMSS-‘Bienestar’ must now confront (Martínez-Valle 2025). Documenting the magnitude and distribution of OOP during this pivotal period provides a baseline against which to judge whether subsequent reforms can improve financial protection for older Mexicans..

Addressing these policy shifts is critical for older adults, who are particularly exposed to the financial consequences of these reforms due both to their health and employment status. Prior analyses using the Mexican Health and Aging Study (MHAS) have demonstrated the prevalence of high OOP in late life linked to frailty, hospitalization, and end-of-life care (Salinas-Escudero et al. 2022). Yet empirical studies examining how older, Mexican adults have fared during this policy transition remain scarce.

Existing analyses of the MHAS data predate the INSABI reform and thus provide an incomplete picture of recent policies. More broadly, Mexico's experience highlights the importance of considering the ageing population in the design of financial protection and the challenges of sustaining this coverage during major health system transitions, a challenge shared by many middle-income countries seeking to expand universal health coverage while controlling OOP spending and ensuring reliable access to medicines and care.

### Study objectives

This study draws on nationally representative data from the 2018 and 2021 waves of the MHAS to offer the first longitudinal analysis of OOP among middle age and older adults following the transition from SP to INSABI. Specifically, we examine the relationship between insurance status, demographic and health characteristics, and OOP across four spending categories: hospitalizations, outpatient procedures, medical visits, and medications. All OOP figures are based on participants’ self-reported spending during the preceding 12 months. Tobit regression models are used to quantify changes in OOP associated with insurance status and the 2019 reform.

This analysis is guided by three hypotheses: (i) Social Security coverage offers the strongest financial protection in both years; (ii) the transition from SP to INSABI is not associated with improved OOP protection; and (iii) older age, female sex, poor self-rated health, and multiple chronic conditions are consistently associated with higher OOP, regardless of insurance status.

## Materials and methods

### Mexican health and aging study

The MHAS is an ongoing, population-based longitudinal cohort study focusing on the ageing population in Mexico. Initiated in 2001, this study includes a representative sample of 15 186 individuals aged 50 and above, as well as their spouses or partners from the same household, regardless of age. Follow-up interviews were conducted in 2003, 2012, 2015, 2018, and 2021. To maintain comprehensive national coverage, new participants aged 50–59 and 50–55, along with their spouses, were specifically recruited in the 2012 and 2018 phases, respectively. The MHAS achieves a consistently high response rate of over 85% across waves, ensuring robust population-level representativeness (Wong et al. 2017).

For our analysis, we used data from the 2018 and 2021 waves of the MHAS, beginning with an initial sample of 14 499 participants across both waves. Of these, we identified individuals who were aged 50 and older in 2018, resulting in 13 684 participants. We then excluded 68 participants with missing information on health insurance, yielding a final sample of 13 616 participants.

### Measures

#### Out-of-pocket expenses

We examined four sources of OOP: hospitalizations, outpatient procedures, medical visits, and medications.

- Hospitalizations: Participants were asked about the number of nights they stayed in a hospital overnight in the last 12 months. Those who reported a hospital stay were then asked about the total amount paid for these hospitalizations.
- Outpatient procedures and medical visits: Participants were asked how often they had outpatient surgeries or visited a doctor in the last 12 months. Those who reported such events were subsequently asked about the total cost of these services.
- Medications: Participants were asked to report the typical monthly cost of their medications over the past 12 months.

Self-reported expenses were initially captured in Mexican Pesos (MXN). For this analysis, we converted these amounts to US Dollars (USD) using the 31 December 2021, exchange rate published by the Bank of Mexico, where 1 USD = 20.5157 MXN (Banco de Mexico 2025b). To compare expenses from 2018 with those from 2021, we adjusted the 2018 values for inflation using the Consumer Price Index (CPI). The inflation factor was calculated by dividing the CPI in 2021 (154.68) by the CPI in 2018 (136.58) (Banco de Mexico 2025a), resulting in an adjustment factor of 1.32. Therefore, all amounts are expressed in 2021 USD.

‘Health Insurance Status’ Participants were asked about their entitlement to medical services under an insurance plan. Those who answered affirmatively were asked to specify their insurance type from a list, which in 2018 included IMSS, ISSSTE, SP, PEMEX, Defence or Marine, Private Medical Insurance, and Other. In 2021, SP was replaced by INSABI.

We categorized participants into five groups: Uninsured; Social Security (IMSS, ISSSTE, PEMEX, Defence, Marine); SP (2018)/INSABI (2021); and Other (private insurance and not specified institutional schemes).

This categorization allows for a clear distinction between public providers based on their financial protection. Options with relatively low response frequencies, such as private medical insurance, were aggregated under ‘Other'.

### Demographic characteristics

We collected information on age (categorized as 50–59, 60–69, 70–79, 80+), sex, marital status (married or in a civil union; single, widowed, separated, or divorced), locality size (≥100 000; <100 000), and education (no schooling; 1–6 years; 7 or more years).

### Health characteristics

- Self-rated health: Participants rated their health as good, fair, or poor.
- Prevalent conditions: Participants were asked at each wave if they had ever been diagnosed by a doctor or medical personnel with diabetes, high blood sugar, hypertension, high blood pressure, stroke, heart failure, heart attack, cancer, asthma, emphysema, or arthritis. A composite variable was created to indicate the presence of one condition, two or more conditions, or the absence of any condition.

### Analysis plan

We examined five outcomes: total OOP expenditures (OOP; primary outcome) and OOP for hospitalizations, outpatient procedures, medical visits, and medications. Each outcome was modelled independently. The primary exposure was health insurance status by year (2018 vs. 2021), distinguishing Social Security, SP/INSABI, Uninsured, and Other. The reference group was Uninsured in 2018.

For each outcome, we first fit a Tobit regression with a lower limit at zero to summarize differences in spending across the full distribution while accounting for the substantial mass at zero and the right-skewed distribution of positive expenditures. All models adjusted for the same set of covariates: age group, sex, marital status, locality size, educational attainment, self-rated health, and a three-level comorbidity count (0, 1, 2+ illnesses). Robust standard errors were used for inference. Because Tobit coefficients are defined on the latent spending scale, we emphasize interpretability by reporting predicted differences in observed OOP in USD relative to the reference group and by presenting average marginal effects. We selected the Tobit model as the primary specification because it provides a single framework for estimating overall differences in spending associated with insurance status and survey year.

However, given that zeros in OOP can reflect true nonuse rather than censoring, we additionally conducted a sensitivity analysis using two-part models (Part 1: logit for any OOP; Part 2: GLM with log link and Gamma family for positive OOP) to assess the robustness of findings from the Tobit regressions. Coefficients from the first-stage coefficients capture factors associated with having any OOP, whereas the second-stage coefficients represent proportional changes in spending among those with positive expenditures (Supplementary Table S3).

In addition, we report unconditional predicted means of OOP unconditional predicted means combining the probability and intensity of spending (Supplementary Table S4). The final results are presented in U.S. dollars as predicted differences versus Uninsured in 2018, with 95% confidence intervals.

Because the study is observational and compares two survey waves before and after a major policy transition, estimates should be interpreted as associations across time and insurance categories rather than causal effects of the reform itself.

## Results

Between 2018 and 2021, during Mexico's transition from SP to INSABI, the proportion of adults aged 50 and older without health insurance nearly tripled, reflecting a sharp decline in financial protection. Simultaneously, OOP expenses remained a persistent burden across health services, with medications consistently representing the largest share of household health spending. These descriptive trends were confirmed in multivariable regression analyses, which showed that uninsurance, older age, female sex, poor self-rated health, and multimorbidity were significantly associated with higher OOP expenditures.

Table 1 summarizes baseline characteristics of the 13 616 participants in 2018. Most were covered by Social Security (59.2%), followed by SP (30.0%), while 9.3% were uninsured. By 2021, Social Security remained the most stable programme, retaining 92.8% of its members; in contrast, more than half of SP beneficiaries lost coverage, and only 31.4% transitioned to INSABI. Among those uninsured in 2018, nearly 70% remained so by 2021, although 21.5% gained Social Security coverage (Supplementary Figure S1).

**Table 1:** Characteristics of the participants in 2018 stratified by insurance type (*N* = 13 616).

	Uninsured	Social Security	‘Seguro Popular’	Other	*P*-value
MHAS participants in 2018	1272	8058	4080	206	
Insurance 2021					<0.001
Uninsured	69.6	5.3	53.1	24.8	
Social Security	21.5	92.8	14.1	37.4	
INSABI	7.5	1.3	31.4	7.3	
Other	1.5	0.5	1.4	30.6	
Utilization in 2018					
Medical visits	45.6	78	68.1	81.1	<0.001
Hospitalizations	5.7	14.1	10.6	17.5	<0.001
Outpatient Procedures	2	5.4	2.9	8.7	<0.001
Age					<0.001
50–59	52.3	35.8	43.8	40.8	
60–69	25	29.6	27.3	24.3	
70–79	15	25.5	20.8	25.2	
80+	7.7	9	8.1	9.7	
Female (versus male)	48.9	58.8	57.7	55.3	<0.001
Marital status					<0.001
Married or in civil union	63.4	69.8	70.5	64.6	
Single, Widowed, Separated or Divorced	36.6	30.2	29.5	35.4	
Locality size					<0.001
≥100 000	52.4	68.5	29.4	63.1	
<100 000 people	47.6	31.5	70.6	36.9	
Employment status					<0.001
Currently working	54.9	39.3	45.5	44.2	
Retired, dedicated to household chores, not working, looking for work	45.1	60.7	54.5	55.8	
Education					<0.001
No formal education	14.6	8.8	22.7	9.8	
1 to 6 years of education	48	43	55	28.9	
7 or more years of education	37.4	48.2	22.3	61.3	
Self-rated health status					<0.001
Excellent, very good, good	42.8	39.8	29.3	51	
Fair	49.9	52.2	59.5	44.3	
Poor	7.3	7.9	11.2	4.7	
Comorbidities					<0.001
No illness	52.5	32.3	38.4	36.9	
1 illness	29.7	35.8	35.5	36.4	
2 + illnesses	17.7	31.9	26.1	26.7	

Values are percentages unless otherwise specified. *P*-values are from Pearson's χ^2^ test for categorical variables. Percentages are column percentages within each insurance type.

Uninsured adults were generally younger and more likely to be employed, while SP affiliates were concentrated in smaller communities. Women were overrepresented in both Social Security and SP. Poor self-rated health was more common among SP participants (11.2%) than in Social Security (7.9%) or the uninsured (7.3%). Multiple chronic conditions were most prevalent in Social Security (31.9%) and least among the uninsured (17.7%).

Healthcare utilization mirrored these disparities: only 45.6% of uninsured respondents reported at least one medical visit in 2018 compared with 78% under Social Security, while hospitalization rates were 5.7% versus 14.1%, respectively.

### Observed out-of-pocket expenses by insurance type

**Figure 1:**
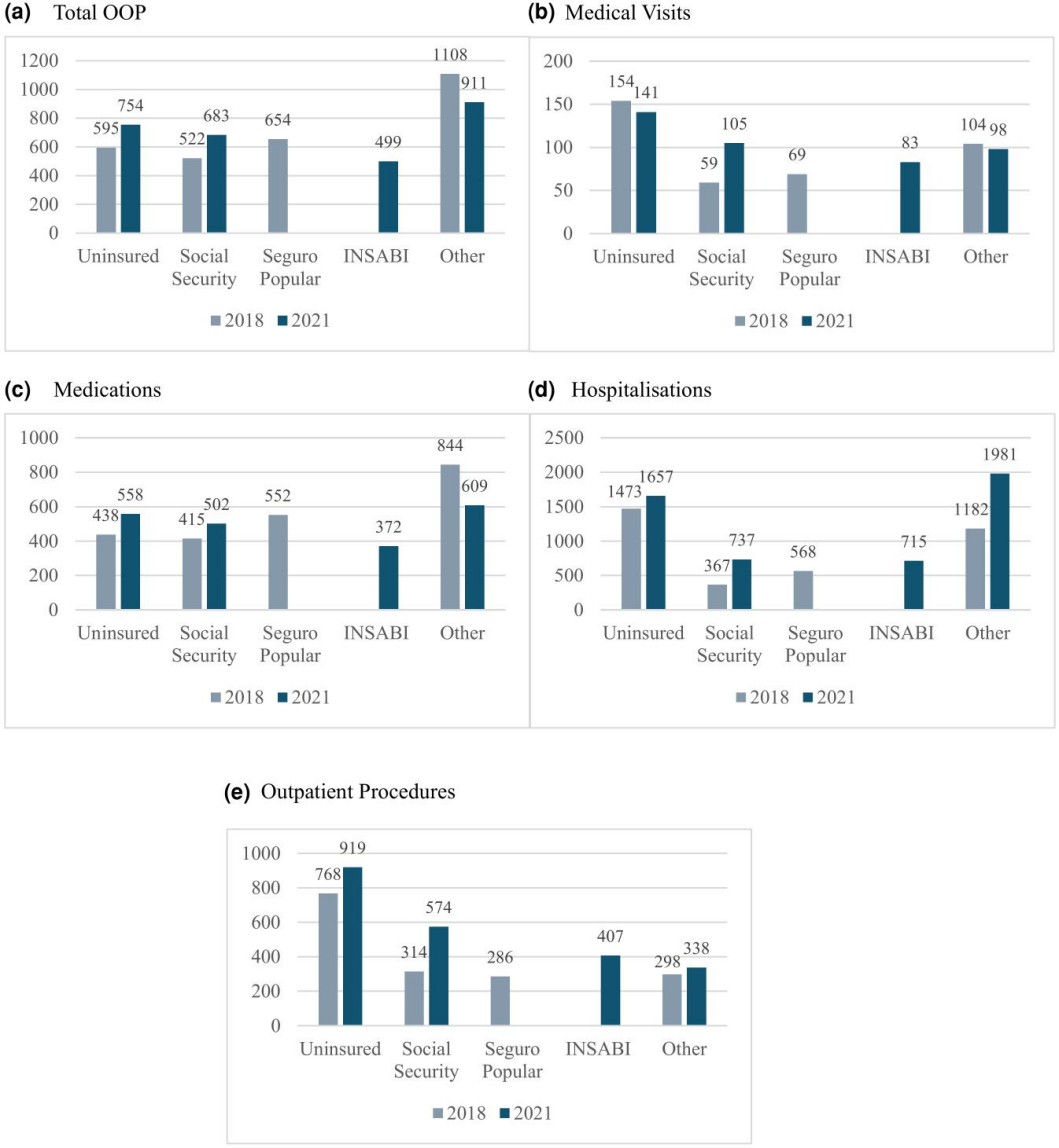
Observed mean out-of-pocket expenditures in 2021 USD by insurance type and spending category among adults aged 50 and older in Mexico, 2018 and 2021. a) Total OOP; b) Medical Visits; c) Medications; d) Hospitalisations; e) Outpatient Procedures

Total observed OOP among the uninsured rose from 595 USD in 2018 to 754 USD in 2021; Social Security participants also experienced an increase (522–683 USD), though at consistently lower levels. Those covered by SP in 2018 spent 654 USD on average, whereas INSABI participants in 2021 paid 499 USD. ‘Other’ insurance, which may include private coverage, had the highest total expenses (1108 USD in 2018), later declining to 911 USD in 2021 (Fig. 1, Supplementary Table S2).

Medical visit spending fell slightly among the uninsured (154–141 USD) but rose for Social Security (59 to 105 USD). ‘Seguro Popular’ participants averaged 69 USD in 2018, and INSABI enrolees reported 83 USD in 2021. The ‘Other’ category remained stable, decreasing marginally from 104 to 98 USD.

Medication spending also varied widely. Among the uninsured, average costs rose from 438 USD in 2018 to 558 USD in 2021, and among Social Security beneficiaries from 415 USD to 502 USD. In contrast, individuals who shifted from SP to INSABI saw their medication OOP fall from 552 USD to 372 USD. The ‘Other’ insurance category likewise declined, dropping from 844 USD to 609 USD.

Hospitalizations accounted for the highest OOP among the uninsured (1473–1657 USD), while Social Security nearly doubled (367–737 USD). ‘Seguro Popular’ participants spent 568 USD, compared to INSABI enrolees at 715 USD. The ‘Other’ group had a marked rise in hospitalization expenses from 1182 to 1981 USD.

For outpatient procedures, the uninsured increased from 768 to 919 USD, and Social Security climbed from 314 to 574 USD. Expenses among SP participants (286 USD) were lower than INSABI (407 USD), while ‘Other’ coverage rose modestly from 298 to 338 USD.

### Tobit regression results

In fully adjusted Tobit models (Table 2), affiliation with Social Security was associated with lower total OOP than being uninsured, but the magnitude of this difference shrank from −1 033 USD (95% CI −1 299 to −768) in 2018 to −539 USD (−795 to −282) in 2021, an attenuation of roughly 495 USD. The coefficient for the group that moved from SP to INSABI remained almost unchanged (−291 USD in 2018 vs −298 USD in 2021), indicating a smaller yet stable spending gap relative to the uninsured. The uninsured in 2021 showed a nonsignificant increase of 218 USD (−43 to 479) compared with the uninsured in 2018, and the ‘Other’ category did not differ significantly in either wave.

**Table 2:** Results of the Tobit regression model estimating total out-of-pocket (OOP) expenses.

	Coefficient	95% CI		*P*-value
Insurance				
Uninsured * 2018	REF			
Uninsured * 2021	217.8	−43.1	478.6	.102
Social Security *2018	**−1033.4**	**−1299.2**	**−767.6**	**<.001**
Social Security * 2021	**−538.7**	**−795.1**	**−282.3**	**<.001**
‘Seguro Popular’ * 2018	**−290.9**	**−564.4**	**−17.4**	**.037**
INSABI * 2021	**−297.7**	**−571.0**	**−24.5**	**.033**
Other * 2018	−576.6	−1320.1	166.8	.128
Other * 2021	−335.5	−872.9	201.9	.221
Age				
50–59	REF			
60–69	−0.4	−132.2	131.4	.995
70–79	**243.6**	**87.1**	**400.1**	**.002**
80+	**559.1**	**292.2**	**826.1**	**<.001**
Female	**322.8**	**190.0**	**455.6**	**<.001**
Marital status				
Married or in civil union	REF			
Single, Widowed, Divorced	−102.8	−227.7	22.1	.107
Locality size				
≥100 000	REF			
<100 000 people	4.9	−111.3	121.1	.934
Employment status				
Currently working	REF			
Retired or not working	116.6	−6.4	239.6	.063
Education				
No formal education	REF			
1 to 6 years	22.6	−172.4	217.6	.820
7 or more years	**647.5**	**428.7**	**866.3**	**<.001**
Self-rated health status				
Excellent	REF			
Fair	**531.9**	**403.2**	**660.6**	**<.001**
Poor	**1257.3**	**990.1**	**1524.6**	**<.001**
Comorbidities				
No illnesses	REF			
1 illness	**702.6**	**564.4**	**840.7**	**<.001**
2 + illnesses	**937.5**	**769.7**	**1105.4**	**<.001**

Coefficients represent predicted differences in OOP, expressed in USD.

REF, reference category. Bold values indicate statistical significance at *P* < .05. *Interaction term. All models control for demographic, socioeconomic, and health-related covariates.

Higher OOP was positively associated with older age, female sex, and multimorbidity. Participants aged 70–79 spent 244 USD more than those aged 50–59 (95% CI 87–400), and those ≥80 years spent 559 USD more (292–826). Women spent 323 USD more than men (190–456). Having ≥7 years of education corresponded to 648 USD higher OOP than having no schooling (429–866). Self-rated fair health added 532 USD (403–661) and poor health 1 257 USD (990–1 525) relative to excellent health. One chronic condition was linked to 703 USD higher spending (564–841) and two or more conditions to 938 USD (770–1 105).

**Figure 2:**
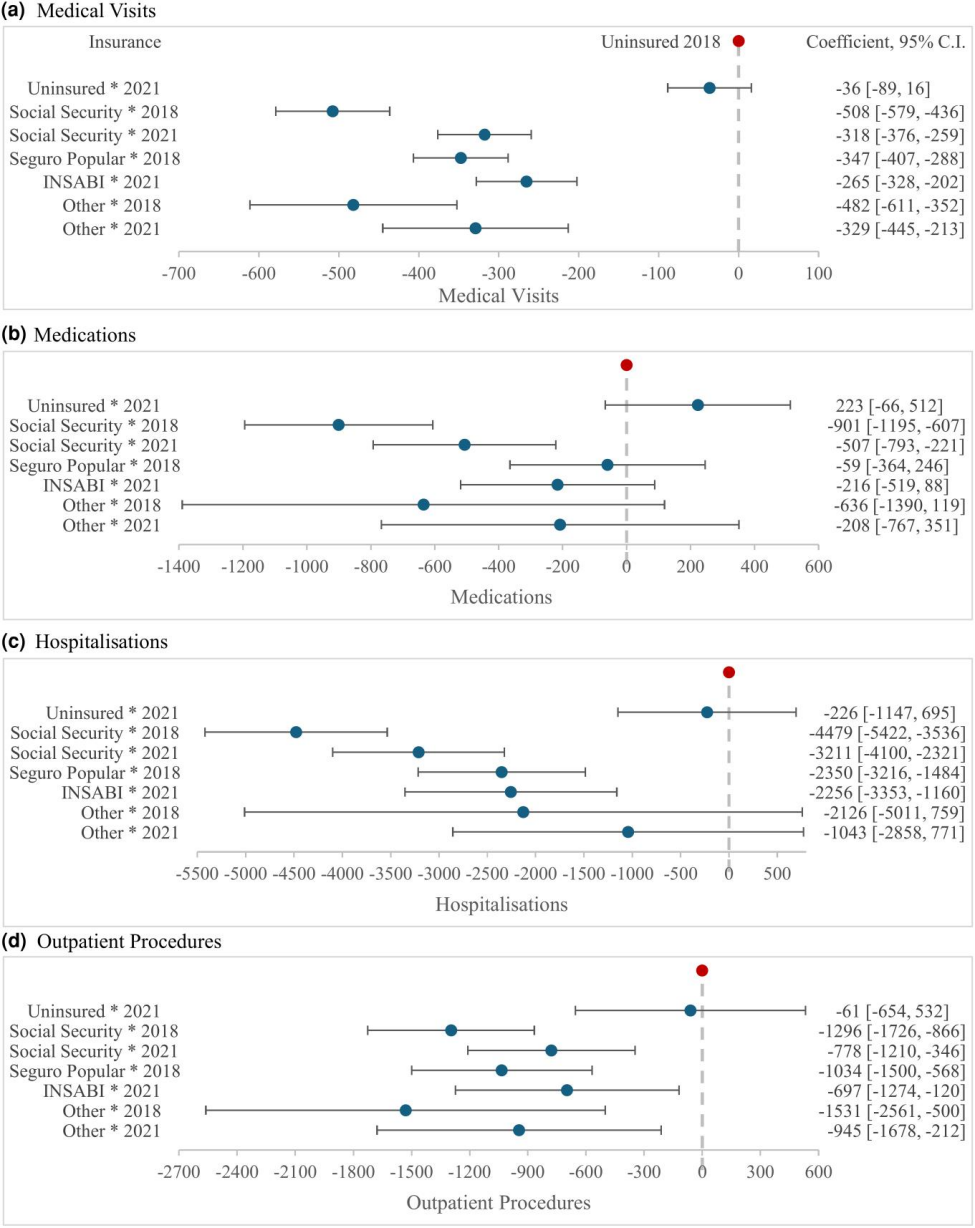
Predicted differences in out-of-pocket expenses (OOP) derived from Tobit regression models, expressed in USD. The reference category is the uninsured population in 2018. All models control for demographic, socioeconomic status, and health-related covariates.

Among medical visits, Social Security was linked to 508 USD lower OOP in 2018 (−579 to −436) and 318 USD lower in 2021 (−376 to −259). The SP/INSABI group showed smaller yet stable differences (−347 USD vs −265 USD). Spending for the uninsured in 2021 (−36 USD; −89 to 16) did not differ significantly from the 2018 uninsured baseline (Fig. 2a).

When analysing expenditures for medications, Social Security was the only insurance mechanism significantly associated with lower spending compared to uninsured, yet the reduction fell from −901 USD (95% CI: −1 195 to −607 USD) in 2018 to −507 USD (95% CI: −793 to −221 USD) in 2021. Coefficients for SP (2018) and INSABI (2021) were negative but not statistically significant, while the uninsured in 2021 trended towards higher, nonsignificant costs at +223 USD (95% CI: −66 to 512 USD) (Fig. 2b).

The largest differences in OOP were observed for hospitalizations: Social Security reduced hospitalization expenses by −4 479 USD (95% CI: −5 422 to −3 536 USD) in 2018 and by −3 211 USD (95% CI: −4 100 to −2 321 USD) in 2021. ‘Seguro Popular’ and its successor INSABI provided significant but smaller relief, −2 350 USD (95% CI: −3 216 to −1 484 USD) and −2 256 USD (95% CI: −3 353 to −1 160 USD), respectively (Fig. 2c).

Protective changes on OOP for outpatient procedures attenuated over time. Social Security cut spending by −1 296 USD (95% CI: −1 726 to −866 USD) in 2018 and −778 USD (95% CI: −1 210 to −346 USD) in 2021. ‘Seguro Popular’ lowered costs by −1 034 USD (95% CI: −1 500 to −568 USD) and INSABI by −697 USD (95% CI: −1 274 to −120 USD). Uninsured participants in 2021 (−61 USD; 95% CI: −654 to 532 USD) showed no meaningful change from the reference group (Fig. 2d).

Overall, the two-part models supported the broader pattern that financial protection was strongest under Social Security and remained limited outside Social Security, but they also revealed important differences from the Tobit models (Supplementary Tables S3 and S4). In the first stage (logit), insurance affiliation was significantly associated with a lower likelihood of incurring any OOP expenditures (OOP), particularly among Social Security beneficiaries compared with uninsured participants in 2018(*β* = −0.76, *P* < 0.001). In the second stage (GLM, log link), conditional on having positive OOP, only INSABI affiliation was associated with significantly lower expenditures (*β* = −0.36, *P* = 0.001) relative to the uninsured. Other insurance types showed no significant differences in conditional amounts.

## Discussion

This study used nationally representative data from the MHAS to assess changes in OOP among adults aged 50 and older during the transition from SP to the INSABI. Across survey waves, Social Security affiliation was consistently associated with the largest reductions in OOP compared to the uninsured, particularly for hospitalizations and medications. The magnitude of this difference declined from −1 033 USD in 2018 to −539 USD in 2021, yet it remained the most pronounced gap observed among all insurance schemes.

Social Security enrolees spent substantially less on medicines (−901 USD in 2018 versus −507 USD in 2021), roughly half the amount reported by uninsured respondents, even during the pandemic years. However, the narrowing of this gap by about 495 USD suggests growing systemic strain within Mexico's health sector, including supply shortages and service disruptions that affected all programs (Martínez-Valle 2025). Despite these pressures, Social Security remains the most reliable source of financial protection and continues to serve as a key benchmark for equity and effectiveness within Mexico's fragmented healthcare landscape. Previous research shows that IMSS-affiliated households had markedly lower risks of catastrophic outlays than uninsured households, largely because they spent less on medicines and hospitalizations (Galárraga et al. 2010). More recent studies show that this financial buffer persisted even during the COVID-19–related disruptions (Serván-Mori et al. 2023).

In contrast, older adults who transitioned from SP to INSABI were associated with only modest and essentially unchanged reductions in total OOP (about −291 USD in 2018 and −298 USD in 2021) and showed no statistically significant difference in medication expenditures. The change in programme affiliation also coincided with a sharp drop in reported coverage: overall enrolment decreased by 53%, and only 31% of former SP beneficiaries reported INSABI coverage, nearly tripling the share of participants reporting no insurance from 9.5% to 27.2%. This pattern suggests that INSABI did not produce a clear improvement in financial protection compared with the pre-existing SP arrangement. At the same time, the lack of statistically significant protection for medication expenditures indicates that both schemes offered incomplete protection in one of the most important spending categories for older adults. This observation is consistent with the National Household Income and Expenditure Survey, which recorded a 39% rise in household spending on medicines between 2018 and 2022, with the poorest households experiencing nearly a two-fold increase (Roldan 2024).

Several underlying systemic factors may explain INSABI's limited capacity to provide financial protection against OOP spending, particularly among older adults. Centralized financing under INSABI has not effectively resolved inefficiencies in regional drug procurement and distribution (Martínez-Valle 2025), leading to recurrent medication shortages that disproportionately affect older adults with chronic and multiple conditions who depend on continuous pharmacological treatment (Rojas Alvarez et al. 2022, García-Hernández et al. 2024, Pérez-Zepeda 2024). When essential medicines are unavailable, older patients are often forced to purchase them from private pharmacies at higher costs, compounding financial strain (Doubova et al. 2024). Furthermore, the removal of formal enrolment procedures blurred patient entitlements, generating confusion about eligibility and leading some public facilities to unofficially reintroduce user fees, an obstacle that tends to weigh most heavily on older adults with limited income or mobility (Doubova et al. 2024, Gómez-Dantés et al. 2024).

Finally, INSABI's fragmented risk pools reduced the potential for effective cross-subsidization across age and income groups, weakening the system's capacity to spread financial risk and undermining governmental bargaining power in bulk drug purchasing (Serván-Mori et al. 2023). These structural deficiencies ultimately expose older Mexicans, who already face higher healthcare utilization, fixed incomes, and polypharmacy, to recurrent OOP costs and a greater likelihood of catastrophic health spending (Rojas Alvarez et al. 2022, Salinas-Escudero et al. 2022).

These patterns are consistent with the segmented design of Mexico's health system. Social security institutions retained their own financing, infrastructure, and entitlement rules after 2020, whereas the reform directly restructured the Ministry of Health services used by people without social security (Gómez-Dantés et al. 2024, Cabrero-Castro et al. 2025). Consequently, older adults dependent on SP/INSABI were more exposed to coverage loss and implementation failures, while social security beneficiaries retained greater institutional continuity.

Among uninsured older adults, there was nonsignificant yet rising averages in both total OOP (+218 USD) and medication spending (+223 USD). These higher costs were more pronounced in groups already linked to greater financial risk: adults aged ≥ 80 years reported 559 USD more OOP, women 323 USD more, and respondents with multimorbidity about 938 USD more than their respective reference groups. Together, these associations highlight the heavier financial load faced by older adults, especially those living on fixed incomes and managing multiple medications (DeGraff et al. 2022, Salinas-Escudero et al. 2022).

Moreover, these findings also highlight a structural weakness in public policy for uninsured older adults in fragmented health systems. In Mexico, financial protection outside social security has historically depended on noncontributory arrangements that have been more vulnerable to redesign, fiscal uncertainty, and uneven implementation than employment-based schemes (Gómez-Dantés et al. 2024). For older adults, this is especially problematic because health needs increase after retirement, while access linked to formal employment becomes less relevant over the life course (Angel et al. 2017).

At the same time, the broader policy context for older adults in Mexico also included an expansion of noncontributory income support. In 2020, a constitutional reform to Article 4 established the right of adults aged 65 years and older to receive a noncontributory state pension. The ‘Pensión para el Bienestar de las Personas Adultas Mayores’ has since operated as a universal cash transfer for this age group (Diario Oficial de la Federación 2020). This programme is especially relevant for older adults without contributory retirement benefits and may have provided some income support during the same period in which SP was dismantled and INSABI was implemented. However, this type of cash transfer should not be interpreted as equivalent to health insurance or direct financial protection against OOP spending. Rather, it may partially mitigate economic vulnerability at the household level while leaving unresolved the specific problems of medicine shortages, unclear entitlements, and weak protection from healthcare costs (Aguila et al. 2018).

A policy architecture that leaves later-life financial protection dependent on unstable noncontributory programs risks reproducing inequities between formally insured and informally employed populations into old age.

The two-part sensitivity analysis adds information to the interpretation of the Tobit results. While the Tobit models summarize overall differences in OOP across the full sample, the two-part models suggest that insurance-related protection may operate mainly through reducing the probability of any spending rather than consistently lowering the amount spent among those with positive expenditures. In particular, in the conditional spending model, only INSABI 2021 was associated with significantly lower OOP relative to the uninsured reference group.

This distinction is substantively important because for older adults, a reduction in the probability of incurring any OOP may reflect improved access to publicly financed care, whereas conditional spending among those who still pay OOP may depend more heavily on service intensity, medicine shortages, or use of private providers.

Taken together, the results focus on two central findings. First, the transition from SP to INSABI coincided with a marked increase in the number of older adults reporting no health insurance, although this finding should be interpreted cautiously given changes in the meaning of self-reported coverage after INSABI eliminated formal enrolment. Second, OOP spending among older adults persisted and, in the case of medications, remained disproportionately high, driving financial burden. These findings point to the fragility of Mexico's health financing reforms and the need to strengthen mechanisms that ensure sustained financial protection, particularly for older populations with chronic health needs.

Beyond its administrative implications, the 2020 reform had important political consequences. The replacement of SP with INSABI represented a politically contentious reorientation of health policy away from a subsidized insurance model and towards a more centralized federal model of service provision (Knaul et al. 2023). Although this shift was presented as a way to expand equity and remove bureaucratic barriers, its implementation coincided with declining reported coverage, ambiguity around entitlement, procurement and medicine supply disruptions, and continued dependence on private ambulatory care (Knaul et al. 2023, Gómez-Dantés et al. 2024, Martínez-Valle 2025). In that sense, the political consequence of the reform was not simply institutional change, but a weakening of already incomplete financial protection for populations outside social security, including many older adults. Mexico's experience therefore illustrates a broader lesson for ageing middle-income countries: reforms framed in universalistic terms can still deepen vulnerability if institutional capacity, financing continuity, and accountability mechanisms are not aligned with the needs of uninsured and informally employed populations.

As Latin America and the Caribbean ages swiftly (in 2022 13.4% of the population were aged 60 years or older, a share projected to rise to 16.5% by 2030), (CEPAL 2022) structural reforms may inadvertently undermine financial protection for older adults unless execution is coordinated and adequately financed. Persistent or rising OOP expenditures during Mexico's transition from SP to INSABI illustrate how reform without strong data systems, medicine supply chains, and integrated delivery can disrupt the financial security of older adults. Similar exposure exists in countries such as Brazil, Colombia and Peru, all facing rapid population ageing, fragmented insurance schemes and high reliance on household health spending (Flores et al. 2025).

The Mexican experience holds broader lessons for ageing societies pursuing universal health coverage under fiscal and institutional constraints. First, it shows that older adults are especially vulnerable to instability in health financing because they require continuous care, regular medication use, and protection that extends beyond formal labour-market participation (World Health Organization 2025a). Second, it underscores that expanding or redesigning coverage on paper does not necessarily translate into financial protection in practice; entitlement clarity, medicine availability, and service delivery capacity are equally critical (Martínez-Valle 2025). Third, in fragmented health systems, reforms that disproportionately affect noncontributory arrangements can widen inequities between populations covered through employment-based insurance and those dependent on government-financed public services (Bancalari et al. 2025). Mexico's transition from SP to INSABI therefore offers a cautionary example for countries attempting to expand universal health coverage while also responding to rapid population ageing and persistent reliance on OOP spending.

### Limitations

First, because the MHAS includes only adults aged 50 and older, our findings are not generalizable to younger populations, and the survey lacks quality-of-care measures. Second, the analysis relies on self-reported expenses, which are subject to recall bias. Third, while Tobit models are widely used to account for the censored nature of health expenditure data, they assume normality and homoscedasticity of errors, which may not fully capture variation in spending. Although we describe coefficients as predicted differences in observed OOP, they technically represent changes in a latent variable. In addition, the study design does not permit causal inference regarding the effects of the SP–INSABI transition. The comparison between 2018 and 2021 spans a period in which multiple concurrent factors may have influenced insurance status, healthcare utilization, and OOP spending, most notably the COVID-19 pandemic and related economic and health system disruptions. Therefore, the observed changes should not be attributed solely to the reform. An additional limitation concerns comparability of self-reported insurance status across waves. In 2021, INSABI operated without formal enrolment, which may have altered how respondents understood and reported coverage relative to SP in 2018. As a result, the category ‘uninsured’ in 2021 may partly reflect uncertainty about entitlement or limited contact with health services rather than complete absence of theoretical eligibility for public care. Because the COVID-19 pandemic also reduced healthcare utilization and public sector contact, changes in insurance status across waves should be interpreted cautiously. Finally, because INSABI was dissolved and replaced by IMSS-‘Bienestar’ in 2023, our findings describe a policy landscape that has already evolved, limiting the direct applicability of these estimates for evaluating the current programme.

### Policy recommendations

To strengthen financial protection for older adults, a phased policy approach is warranted. Short-term actions should ensure uninterrupted medicine supply within ‘IMSS-Bienestar’ by strengthening pharmaceutical logistics, transparency in procurement, and age-prioritized drug distribution. Medium-term reforms should enhance system equity by pooling pharmaceutical purchasing across insurance schemes, implementing bundled payments for chronic disease management, and scaling effective elements of the social security model to populations without formal employment. Long-term strategies must prioritize a transition to a unified, employment-independent financing model that guarantees coverage continuity into old age.

Pharmaceutical coverage should remain central to this reform agenda. For ageing populations, decoupling financial protection from formal labour status is essential to prevent recurrent financial shocks during retirement. Policies should incorporate chronic disease medication subsidies, essential-drug delivery to rural areas, and age-adapted benefit packages that address polypharmacy and long-term care needs.

Finally, governments across Latin America and other rapidly ageing middle-income regions should integrate health financing and service delivery to reduce fragmentation and enhance accountability. Expanding pooled funding mechanisms, securing essential medicine supply chains, and investing in data systems to monitor older adults’ access and financial protection are critical steps to ensure that future reforms, whether in Mexico or elsewhere, advance universal health coverage while safeguarding older populations from catastrophic health spending.

## Conclusion

Mexico's rapidly ageing population continues to face substantial OOP burdens, particularly for medicines in the absence of robust and inclusive health insurance mechanisms. Older adults, who experience the highest prevalence of multimorbidity and rely heavily on medications for chronic disease management, remain disproportionately exposed to these financial pressures.

The transition from SP to INSABI coincided with a marked increase in the proportion of older adults reporting no health insurance, although this finding should be interpreted cautiously given changes in the meaning of self-reported coverage after INSABI eliminated formal enrolment. OOP remained high throughout the study period, with pharmaceuticals continuing to be the principal source of financial burden among older Mexicans. While Social Security consistently provided the strongest financial protection, SP in 2018 and INSABI in 2021 showed only modest and broadly similar reductions in OOP relative to the uninsured. Overall, the findings indicate no clear improvement in financial protection for older adults outside Social Security and highlight the continuing fragility of health financing arrangements for ageing populations with substantial chronic care needs. Ensuring effective financial protection for older adults will require policies that strengthen pharmaceutical coverage, clarify entitlement pathways, and harmonize benefits across fragmented insurance schemes.

More broadly, Mexico's experience illustrates a wider regional challenge: sustaining financial protection during rapid population ageing requires not only expanding coverage but also building resilient institutions capable of delivering equitable, efficient, and reliable care amid fiscal and demographic constraints. Future research should evaluate the implications of the IMSS-Bienestar transition using longitudinal data, assessing changes in OOP, foregone care, and quality of chronic disease management among older adults. Comparative studies across ageing middle-income countries in Latin America could further illuminate how institutional design shapes the capacity to protect older populations from medical impoverishment.

## Supplementary Material

czag059_Supplementary_Data

## Data Availability

All data files used in this study are publicly available upon registration for download at www.mhasweb.org.

## Reflexivity statement

The author team comprises three women and four men and spans multiple career stages, with three early-career researchers and four senior academics. Three of the authors are economists, and as a group we bring interdisciplinary expertise in the quantitative analysis of health policy in low- and middle-income countries, with sustained research engagement in Mexico. All authors have experience analysing Mexican health policies and reforms, particularly those affecting financial protection and access to care for older adults.
