# Supplementary material for: Financial protection in flux: longitudinal evidence on out-of-pocket spending among middle-aged and older Mexicans during the ‘Seguro Popular’–INSABI transition
Source: Health Policy Plan. 2026 May 7;41(7):1067–79. doi: 10.1093/heapol/czag059 (PMC13481689; doi:10.1093/heapol/czag059)
Supplement: Supplementary file 1 [file czag059_supplementary_data.zip › Supplementary material INSABI OOP R1 clean version.docx]

**Supplementary material**

**Financial Protection in Flux: Longitudinal Evidence on Out-of-Pocket Spending Among Middle-Aged and Older Mexicans During the *Seguro Popular*–INSABI Transition**

**Table of Contents**

1. **Figure S1. Flow of Health Insurance Coverage between 2018 and 2021 in the Mexican Health and Aging Study.**
2. **Table S1. Dictionary with MHAS question references, coding and brief notes.**
3. **Table S2. Observed out-of-pocket expenditures (OOP) by insurance type in 2018 and 2021.**
4. **Table S3. Two-Part Model of Out-of-Pocket Expenditures (OOP)**
5. **Table S4. Summary table of predicted mean OOP (USD) from two-part model.**

**Figure S1. Flow of Health Insurance Coverage between 2018 and 2021 in the Mexican Health and Aging Study. The width of each band is proportional to the number of individuals moving from a given 2018 insurance category to a 2021 category. (N = 13,616)**

**Table S1. Dictionary with MHAS question references, coding and brief notes.**

Panel A. Outcomes (OOP) — coding and MHAS source

| **Variable** | **Coding / Unit** | **MHAS item (question)** | **Wording (abridged)** | **Notes** |
| --- | --- | --- | --- | --- |
| **Total OOP** | Sum of 4 components (USD, 2021-adjusted) | Derived | — | Sum of medications + consultations + hospitalizations + outpatient procedures; 2018 amounts CPI-adjusted to 2021, then converted to USD. |
| **OOP—Medications** | Continuous (currency → USD) | **D.12a** | “In the last year, in a typical month how much was paid for the medications you take?” | Multiply monthly by 12. |
| **OOP—Consultations** | Continuous (currency → USD) | **D.9** | “Including all visits, how much did you pay for these services?” | Use the consultations subitem of D.9. Annual period. |
| **OOP—Hospitalizations** | Continuous (currency → USD) | **D.6** | “Altogether, how much did you pay for these hospitalizations?” | Annual period. |
| **OOP—Outpatient procedures** | Continuous (currency → USD) | **D.9** | “Including all visits, how much did you pay for these services?” | Use the outpatient-procedures subitem of D.9. Annual period. |

Panel B. Main predictor and covariates – coding and MHAS source

| **Variable** | **Coding** | **MHAS item (question)** | **Wording (abridged)** | **Notes** |
| --- | --- | --- | --- | --- |
| **Predictor** |  |  |  |  |
| **Health insurance (2018/2021)** | **2018 categories:** 1 Uninsured; 2 Social Security (IMSS/ISSSTE/PEMEX/SEDENA/SEMAR); 3 *Seguro Popular*; 4 Other. **2021 categories:** 1 Uninsured; 2 Social Security; 3 INSABI; 4 Other. | **D.1** | “Do you have the right to medical service in…?” | Interaction term Insurance×Year used. |
| **Covariates** |  |  |  |  |
| **Age group** | 1 50–59; 2 60–69; 3 70–79; 4 80+ | **A.2b** | “How old are you in full years?” | Derived from age in years at interview; grouped as shown. |
| **Sex** | 1 Male; 2 Female | **A.1** | “INTERVIEWER: Indicate respondent’s sex.” | — |
| **Marital status** | 1 Married or in civil union; 2 Single/Widowed/Separated/Divorced | **A.3** | “Currently are you…?” | Two-level recode. |
| **Locality size** | 1 ≥100,000 inhabitants; 2 <100,000 inhabitants | **Contextual (N/A)** | — | From MHAS contextual link to INEGI census; not self-reported. |
| **Employment status** | 1 Currently working; 2 Not working/retired/household/looking for work | **I.16** (currently working) and **I.26** (reason not working) | “Currently you… / What is the reason you do not work?” | Collapsed to two levels. |
| **Educational attainment** | 1 No formal education; 2 1–6 years; 3 ≥7 years | **AA.4a** | “What is the last year/grade completed in school?” | Years of schooling recoded to categories. |
| **Self-rated health** | 1 Excellent/Very good/Good; 2 Fair; 3 Poor | **C.1** | “Would you say your health is…?” | Three-level recode. |
| **Chronic conditions (count)** | 0 None; 1 One; 2 Two or more | **C.4** Hypertension; **C.6** Diabetes; **C.12** Cancer; **C.19** Respiratory illness (e.g., asthma/emphysema); **C.22a** Heart attack; **C.26** Stroke/TIA; **C.32** Arthritis; **C.25b** Cardiac problems | “Has a doctor ever diagnosed you with…?” | Sum of listed physician-diagnosed conditions; recoded to 0/1/2+. |

**Table S2. Observed out-of-pocket expenditures (OOP) by insurance type in 2018 and 2021.**

| **Insurance type** | **2018**  **Observed OOP** | **SD** | **2021**  **Observed OOP** | **SD** |
| --- | --- | --- | --- | --- |
| **Total** |  |  |  |  |
| Uninsured | 595 | 2643 | 754 | 2961 |
| Social Security | 522 | 3196 | 683 | 2557 |
| *Seguro Popular* | 654 | 4031 | — | — |
| INSABI | — | — | 499 | 1579 |
| Other | 1108 | 5784 | 911 | 2854 |
| **Hospitalizations** |  |  |  |  |
| Uninsured | 1473 | 2262 | 1657 | 2701 |
| Social Security | 367 | 1391 | 737 | 2021 |
| *Seguro Popular* | 568 | 1286 | — | — |
| INSABI | — | — | 715 | 1760 |
| Other | 1182 | 4119 | 1981 | 4065 |
| **Medications** |  |  |  |  |
| Uninsured | 438 | 2456 | 558 | 2700 |
| Social Security | 415 | 3098 | 502 | 2289 |
| *Seguro Popular* | 552 | 3994 | — | — |
| INSABI | — | — | 372 | 1357 |
| Other | 844 | 5225 | 609 | 1580 |
| **Medical Visits** |  |  |  |  |
| Uninsured | 154 | 456 | 141 | 456 |
| Social Security | 59 | 323 | 105 | 516 |
| *Seguro Popular* | 69 | 394 | — | — |
| INSABI | — | — | 83 | 260 |
| Other | 104 | 485 | 98 | 238 |
| **Outpatient Procedures** |  |  |  |  |
| Uninsured | 768 | 932 | 919 | 1537 |
| Social Security | 314 | 850 | 574 | 882 |
| *Seguro Popular* | 286 | 657 | — | — |
| INSABI | — | — | 407 | 905 |
| Other | 298 | 696 | 338 | 430 |

Values are observed means in USD with standard deviations (SD).

**Table S3. Two-Part Model of Out-of-Pocket Expenditures (OOP)**

| **Variable** | **Coefficient** | **p-value** | **95 % CI** |
| --- | --- | --- | --- |
| **Part 1 – Logit: Probability of any OOP** |  |  |  |
| Uninsured * 2018 | REF |  |  |
| Social Security 2018 vs Uninsured 2018 | **-0.757** | **< 0.001** | −0.886 , −0.629 |
| Seguro Popular 2018 vs Uninsured 2018 | **-0.206** | **0.003** | −0.342 , −0.070 |
| INSABI 2021 vs Uninsured 2018 | **-0.242** | **0.001** | −0.379 , −0.106 |
| Other 2018 vs Uninsured 2018 | **-0.696** | **< 0.001** | −1.018 , −0.373 |
| Uninsured 2021 vs Uninsured 2018 | **0.249** | **< 0.001** | +0.109 , +0.389 |
| Additional difference for Social Security 20211 | 0.109 | 0.167 | −0.046 , +0.263 |
| Additional difference for Other 2021 | 0.125 | 0.592 | −0.331 , +0.580 |
| Age 60-69 | -0.056 | 0.104 | -0.123, +0.011 |
| Age 70–79 | 0.067 | 0.087 | −0.010 , +0.144 |
| Age 80+ | **0.124** | **0.026** | +0.015 , +0.234 |
| Female | **0.270** | **< 0.001** | +0.210 , +0.329 |
| Married/Union | **0.014** | **0.647** | -0.046, +0.073 |
| Locality <100,000 | 0.009 | 0.760 | -0.048, +0.066 |
| Retired/Not working | -0.006 | 0.838 | -0.068, +0.056 |
| 1–6 years education | **0.059** | **0.165** | -0.024, +0.143 |
| ≥ 7 yrs education | **0.403** | **< 0.001** | +0.309 , +0.496 |
| Fair health | **0.302** | **< 0.001** | +0.244 , +0.360 |
| Poor health | **0.644** | **< 0.001** | +0.536 , +0.752 |
| 1 illness | **0.506** | **< 0.001** | +0.444 , +0.568 |
| 2 + illnesses | **0.556** | **< 0.001** | +0.486 , +0.627 |
| Constant | **-0.223** | **0.005** | −0.378 , −0.069 |
| **Part 2 – GLM (Gamma–log): Conditional OOP > 0** |  |  |  |
| Uninsured * 2018 | REF |  |  |
| Social Security 2018 vs Uninsured 2018 | -0.041 | 0.780 | −0.327 , +0.245 |
| Seguro Popular 2018 vs Uninsured 2018 | -0.010 | 0.950 | −0.333 , +0.312 |
| INSABI 2021 vs Uninsured 2018 | **-0.357** | **0.001** | −0.565 , −0.149 |
| Other 2018 vs Uninsured 2018 | 0.339 | 0.212 | −0.194 , +0.872 |
| Uninsured 2021 vs Uninsured 2018 | -0.028 | 0.849 | −0.312 , +0.256 |
| Additional difference for Social Security 20211 | 0.045 | 0.786 | -0.279, +0.369 |
| Additional difference for Other 2021 | -0.197 | 0.547 | -0.839, +0.444 |
| Age 60-69 | 0.090 | 0.265 | -0.069, +0.249 |
| Age 70–79 | **0.201** | **0.020** | +0.032 , +0.370 |
| Age 80+ | **0.468** | **< 0.001** | +0.227 , +0.708 |
| Female | -0.018 | 0.791 | -0.153, +0.117 |
| Married/Union | **-0.163** | **0.021** | −0.300 , −0.025 |
| Locality <100,000 | -0.046 | 0.455 | -0.167, +0.075 |
| Retired/Not working | **0.200** | **0.006** | +0.058 , +0.341 |
| 1–6 years education | -0.071 | 0.520 | -0.286, +0.145 |
| ≥ 7 yrs education | 0.182 | 0.116 | -0.045, +0.409 |
| Fair health | **0.257** | **< 0.001** | +0.125 , +0.390 |
| Poor health | **0.593** | **< 0.001** | +0.399 , +0.787 |
| 1 illness | **0.117** | **0.116** | -0.029, +0.262 |
| 2 + illnesses | **0.343** | **< 0.001** | +0.185 , +0.502 |
| Constant | **6.349** | **< 0.001** | +5.989, +6.709 |

Dependent variable: OOP (USD).

Part 1 = logit (probability of any OOP); Part 2 = GLM Gamma-log (conditional amount).

Reference category: *Uninsured × 2018.*

In the two-part model, the coefficient for Uninsured 2021 represents the contrast between uninsured respondents in 2021 and uninsured respondents in 2018. Insurance coefficients represent contrasts versus Uninsured 2018 in the baseline year, except for INSABI, which was observed only in 2021. Interaction terms represent the additional 2021 deviation for categories observed in both years (Social Security and Other). Because Seguro Popular was observed only in 2018 and INSABI only in 2021, the insurance-by-year parameterization is not fully symmetric across categories.

**Table S4. Summary table of predicted mean OOP (USD) from two-part model.**

| **Insurance Type** | **2018 (Mean, USD)** | **95% CI** | **2021 (Mean, USD)** | **95% CI** | **Change (2021–2018)** |
| --- | --- | --- | --- | --- | --- |
| **Uninsured** | 692.7 | (516.4 – 869.0) | **723.1** | (623.2 – 823.0) | **30.4** |
| **Social Security** | 495.5 | (428.2 – 562.9) | **588.5** | (535.1 – 641.8) | **92.9** |
| **Seguro Popular 2018 / INSABI 2021** | 640.7 | (511.4 – 770.0) | **472.5** | (390.6 – 554.4) | **-168.2** |
| **Other** | 745.8 | (381.4 – 1110.1) | **695.8** | (453.2 – 938.4) | **–50.0** |

Predicted unconditional mean OOP (USD) by *insurance type × year (2018 vs 2021)*, combining both the probability of spending (logit) and the level of spending among those who did (GLM Gamma-log).
