# Supplementary material for: Financial protection in flux: longitudinal evidence on out-of-pocket spending among middle-aged and older Mexicans during the ‘Seguro Popular’–INSABI transition
Source: Health Policy Plan. 2026 May 7;41(7):1067–79. doi: 10.1093/heapol/czag059 (PMC13481689; doi:10.1093/heapol/czag059)
Supplement: Supplementary file 1 [file czag059_supplementary_data.zip › Tables OOP HP&P.docx]

**Table 1. Characteristics of the participants in 2018 stratified by insurance type (N=13,616).**

|  | | Uninsured | Social Security | *Seguro Popular* | Other | p-value |
| --- | --- | --- | --- | --- | --- | --- |
| MHAS Participants in 2018 |  | 1,272 | 8,058 | 4,080 | 206 |  |
|  |  |  |  |  |  |  |
| Insurance 2021 | |  |  |  |  | <0.001 |
|  | Uninsured | 69.6 | 5.3 | 53.1 | 24.8 |  |
|  | Social Security | 21.5 | 92.8 | 14.1 | 37.4 |  |
|  | INSABI | 7.5 | 1.3 | 31.4 | 7.3 |  |
|  | Other | 1.5 | 0.5 | 1.4 | 30.6 |  |
| Utilization in 2018 | |  |  |  |  |  |
|  | Medical Visits | 45.6 | 78 | 68.1 | 81.1 | <0.001 |
|  | Hospitalizations | 5.7 | 14.1 | 10.6 | 17.5 | <0.001 |
|  | Outpatient Procedures | 2 | 5.4 | 2.9 | 8.7 | <0.001 |
| Age |  |  |  |  |  | <0.001 |
|  | 50-59 | 52.3 | 35.8 | 43.8 | 40.8 |  |
|  | 60-69 | 25 | 29.6 | 27.3 | 24.3 |  |
|  | 70-79 | 15 | 25.5 | 20.8 | 25.2 |  |
|  | 80+ | 7.7 | 9 | 8.1 | 9.7 |  |
|  | |  |  |  |  |  |
| Female (vs. Male) |  | 48.9 | 58.8 | 57.7 | 55.3 | <0.001 |
| Marital status | |  |  |  |  | <0.001 |
|  | Married or in civil union | 63.4 | 69.8 | 70.5 | 64.6 |  |
|  | Single, Widowed, Separated or Divorced | 36.6 | 30.2 | 29.5 | 35.4 |  |
| Locality size | |  |  |  |  | <0.001 |
|  | >=100,000 | 52.4 | 68.5 | 29.4 | 63.1 |  |
|  | <100,000 people | 47.6 | 31.5 | 70.6 | 36.9 |  |
| Employment status | |  |  |  |  | <0.001 |
|  | Currently working | 54.9 | 39.3 | 45.5 | 44.2 |  |
|  | Retired, dedicated to household chores, not working, looking for work | 45.1 | 60.7 | 54.5 | 55.8 |  |
| Education | |  |  |  |  | <0.001 |
|  | No formal education | 14.6 | 8.8 | 22.7 | 9.8 |  |
|  | 1 to 6 years of education | 48 | 43 | 55 | 28.9 |  |
|  | 7 or more years of education | 37.4 | 48.2 | 22.3 | 61.3 |  |
| Self-rated health status | | |  |  |  | <0.001 |
|  | Excellent, very good, good | 42.8 | 39.8 | 29.3 | 51 |  |
|  | Fair | 49.9 | 52.2 | 59.5 | 44.3 |  |
|  | Poor | 7.3 | 7.9 | 11.2 | 4.7 |  |
| Comorbidities | |  |  |  |  | <0.001 |
|  | No illness | 52.5 | 32.3 | 38.4 | 36.9 |  |
|  | 1 illness | 29.7 | 35.8 | 35.5 | 36.4 |  |
|  | 2+ illnesses | 17.7 | 31.9 | 26.1 | 26.7 |  |

Values are percentages unless otherwise specified. p-values are from Pearson’s χ² test for categorical variables. Percentages are column percentages within each insurance type.

**Table 2. Results of the Tobit regression model estimating total out-of-pocket expenses (OOP). Coefficients represent predicted differences in OOP, expressed in USD.**

| Insurance | | Coefficient | 95% CI | | p-value |
| --- | --- | --- | --- | --- | --- |
|  | Uninsured * 2018 | REF |  |  |  |
|  | Uninsured * 2021 | 217.8 | -43.1 | 478.6 | 0.102 |
|  | Social Security *2018 | **-1033.4** | **-1299.2** | **-767.6** | **<0.001** |
|  | Social Security * 2021 | **-538.7** | **-795.1** | **-282.3** | **<0.001** |
|  | *Seguro Popular* * 2018 | **-290.9** | **-564.4** | **-17.4** | **0.037** |
|  | INSABI * 2021 | **-297.7** | **-571.0** | **-24.5** | **0.033** |
|  | Other * 2018 | -576.6 | -1320.1 | 166.8 | 0.128 |
|  | Other * 2021 | -335.5 | -872.9 | 201.9 | 0.221 |
|  |  |  |  |  |  |
| Age |  |  |  |  |  |
|  | 50-59 | REF |  |  |  |
|  | 60-69 | -0.4 | -132.2 | 131.4 | 0.995 |
|  | 70-79 | **243.6** | **87.1** | **400.1** | **0.002** |
|  | 80+ | **559.1** | **292.2** | **826.1** | **<0.001** |
|  |  |  |  |  |  |
| Female |  | **322.8** | **190.0** | **455.6** | **<0.001** |
| Marital status | |  |  |  |  |
|  | Married or in civil union | REF |  |  |  |
|  | Single, Widowed, Divorced | -102.8 | -227.7 | 22.1 | 0.107 |
| Locality size | |  |  |  |  |
|  | >=100000 | REF |  |  |  |
|  | <100,000 people | 4.9 | -111.3 | 121.1 | 0.934 |
| Employment status | |  |  |  |  |
|  | Currently working | REF |  |  |  |
|  | Retired or not working | 116.6 | -6.4 | 239.6 | 0.063 |
| Education | |  |  |  |  |
|  | No formal education | REF |  |  |  |
|  | 1 to 6 years | 22.6 | -172.4 | 217.6 | 0.820 |
|  | 7 or more years | **647.5** | **428.7** | **866.3** | **<0.001** |
| Self-rated health status | | |  |  |  |
|  | Excellent | REF |  |  |  |
|  | Fair | **531.9** | **403.2** | **660.6** | **<0.001** |
|  | Poor | **1257.3** | **990.1** | **1524.6** | **<0.001** |
| Comorbidities | |  |  |  |  |
|  | No illnesses | REF |  |  |  |
|  | 1 illness | **702.6** | **564.4** | **840.7** | **<0.001** |
|  | 2+ illnesses | **937.5** | **769.7** | **1105.4** | **<0.001** |

REF = Reference category. Bold values indicate statistical significance at *p* < 0.05. All models control for demographic, socioeconomic, and health-related covariates.
